# Moving Ahead: What’s Next for the eGEMs Community

**DOI:** 10.5334/egems.313

**Published:** 2019-05-16

**Authors:** Paul Wallace

**Affiliations:** 1 AcademyHealth, US

**Keywords:** electronic health data, EHD, health informatics, learning health systems

## Abstract

*eGEMs*, in close partnership with our key sponsor and publisher, AcademyHealth, has provided a window to the transformational impact of electronic health data (EHD) on how we pursue health and deliver healthcare. This commentary traces key milestones in that journey and announces the next chapter for this community and the critical work it produces.

In 2010, the Agency for Healthcare Research and Quality (AHRQ) began active funding of research projects in the use of EHD, followed quickly in 2011 by the establishment of the Electronic Data Methods (EDM) Forum with coordination through AcademyHealth. As the EDM Forum attracted novel ideas and offered collaboration opportunities, it grew over 2 to 3 years from a few hundred active researchers to several thousand engaged investigators, sponsors, end users, and stakeholders [1].

To further support this emerging community, in January 2013 as part of the EDM Forum grant, *eGEMs* began publication as an open-source, peer reviewed online journal. Three commentaries among the initial papers noted the opportunity of that moment:

- Lisa Simpson, the CEO of AcademyHealth wrote: *Major advances in the availability of electronic clinical data (ECD) provide the opportunity to address questions that are important to the recipients, providers, and purchasers of health care; …As the professional society for the field, AcademyHealth supports innovation, adaptation, and learning to advance research itself. We are launching eGEMs in response to the expressed needs of our members and partners as part of helping our field “learn how to learn” and capitalize on the opportunities in the present research environment* [2].
- Erin Holve, the Project Director for the EDM Forum and the inaugural Executive Editor of *eGEMs* noted: *This focus is an effort to highlight innovative approaches that are advancing our knowledge on both technical dimensions (analytic methods; informatics) and non-technical dimensions (governance; achieving the goals of a learning health system that can improve patient outcomes) for the conduct of CER, PCOR, and QI* [3].
- I added: *In order to achieve its goals, eGEMs should aim to promote rapid sharing of ideas, engage sponsors and potential users of research findings early in the process, develop metrics for success to guide research efforts, and recruit diverse contributors. Success would allow for not just the generation of new research, but also new science, which has the potential to significantly improve patient outcomes* [4].

These interacting themes of building community, learning how to learn collaboratively and faster, and seeking innovative and novel science have sustained the engagement of the researchers and knowledge seekers involved with this journal.

A lot happens over 6 years! Progress with using EHD has accelerated and expanded the integration of EHD and advanced analytics into clinical and operational decision making. *eGEMs* has actively contributed to this body of understanding. Between 2013 and 2019, *eGEMs* will have published over 260 papers featuring the work of more than 900 authors from over 500 institutions. *eGEMs* papers have been viewed or downloaded thousands of times—both from the journal’s own platform and from PubMed Central (PMC)—and have been cited hundreds of times by other researchers. Since its inception, the topic areas in *eGEMs* published papers have also expanded. The most viewed papers since the end of the EDM Forum grant in March 2017 reflects high interest in delivery system content including a special issue [5] on key learnings from the High Value Healthcare Collaborative as well as a series of papers on analytic methods for learning health systems (see Figure 1). Click here to access an interactive version of Figure 1, where you can hover over each bubble for complete paper titles, lead author name, volume and issue number, and number of views.

**Figure 1:**
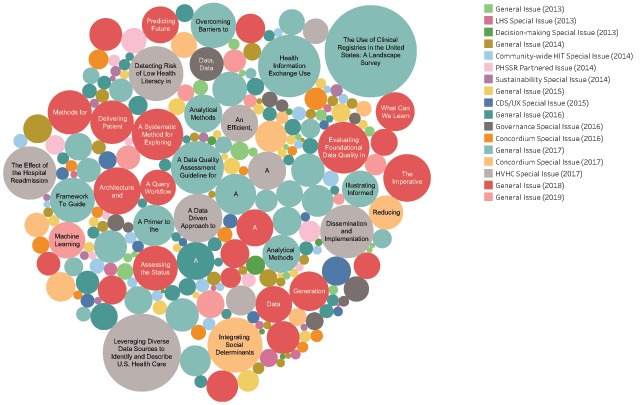
eGEMs Most Viewed Papers.

A few weeks ago, *eGEMs* published a substantial special issue [6] of 16 articles focused again on health care delivery. The issue commemorates 25 years of the Health Care Systems Research Network’s (HCSRN) rigorous research to improve health outcomes and health systems’ performance by leveraging electronic health data. The issue’s papers highlight innovative use of electronic health data to address a variety of health care delivery improvements including personalized medicine, data sharing for comparative health system performance, use of self-reported data, and data quality assessment. Findings in the collection have implications for cancer care, suicide prevention, pediatric care in emergency room settings, chronic disease clinical decision support, and more. Still to come next month, *eGEMs* will publish another special issue showcasing research that leverages data as a driver of health system transformation. Sponsored by AHRQ, the publication will highlight new data insights and capabilities that respond to rapid change in the way researchers, clinicians, patients, policymakers and others use data to transform health care.

At its core, scholarship is intended to foster change, and as the *eGEMs* community can attest, the research and learning environment is shifting rapidly. Systematic learning is increasingly organized around, and studies done, within systems of care delivery. Further, the linkage that *eGEMs* was created to fill is now morphing into a networked community of data users and customers. Their experiences are being synthesized and shared through forums from social media to Health Datapalooza. Finally, the work of the data community is increasingly sought by both long established and more recently developed peer-reviewed publications.

I’m pleased that AcademyHealth is at the forefront in building the learning healthcare ecosystem, through strategic investments as well as sustaining and building partnerships to realize opportunity. Under the stewardship of AcademyHealth, *eGEMs* has also transitioned from its origin as a grant supported journal to seeking self-support through sponsorships and article processing fees provided by authors. Now, given the advances in integration of health data methods into the wider field of delivery system science, AcademyHealth has partnered with the Elsevier journal *Healthcare: The Journal of Delivery Science and Innovation*, to debut a new publishing home for the critical work published in *eGEMs*. *Insights in Analyzing Health Data* is an ongoing section dedicated to data methods and applications within Healthcare and guided by select members of the *eGEMs* editorial board.

In testing the possibilities with AcademyHealth staff, several key *eGEMs* stakeholders, plus the editors of *Healthcare*, we feel that this is an excellent opportunity to sustain the commitment to the *eGEMs* community while also allowing AcademyHealth to turn its attention to other areas of the health care delivery research community where gaps remain.

We anticipate the final papers will publish in June on the *eGEMs* platform, but the legacy of *eGEMs* will be preserved. All *eGEMs* published papers will maintain their original DOIs and continue to be freely available through archiving on CLOCKSS and through PMC. Further, *Healthcare* has become an official journal of AcademyHealth offering both subscription and article processing charge discounts to AcademyHealth members as well as partnering with AcademyHealth to ensure work published in the journal reaches a broad spectrum of health services, policy and system researchers, practitioners and funders.

In summary, as a desirable and innovative solution, I see this path forward as hitting the “both/and” of ongoing service to the *eGEMs* community and adapting to this dynamic health data ecosystem.

Finally, as Executive Editor, I’d like to extend my deep appreciation to our authors and readers, the volunteer editorial board and peer-reviewers, the original funders from AHRQ, special issue sponsors (AHRQ, Intermountain Healthcare, and the HCSRN), and the AcademyHealth leadership, staff and Board of Directors. A special thanks to Lauren Adams and David Padgham for their close partnership during my tenure with *eGEMs*. We look forward to seeing more published work from this community in *Insights for Analyzing Health Data*!

*To learn more about this next chapter of the eGEMs community and for answers to frequently asked questions about this transition, please see the AcademyHealth website here*.
